# Comparison between magnetic resonance imaging and electrical impedance myography for evaluating lumbar skeletal muscle composition

**DOI:** 10.1186/s12891-022-05902-9

**Published:** 2022-11-09

**Authors:** Domenico Albano, Salvatore Gitto, Jacopo Vitale, Susan Bernareggi, Alberto Aliprandi, Luca Maria Sconfienza, Carmelo Messina

**Affiliations:** 1 IRCCS Istituto Ortopedico Galeazzi, Milan, Italy; 2 Dipartimento di Scienze Biomediche per la Salute, Università degli Studi di Milano, Milan, Italy; 3 Unit of Radiology, Clinical Institutes Zucchi, Monza, Monza Brianza Italy

**Keywords:** Body fat percentage, Skulpt chisel™, Electrical impedance myography, Magnetic resonance imaging, Body composition, Sarcopenia, Lumbar spine

## Abstract

**Background:**

To compare electrical impedance myography (EIM) and MRI in assessing lumbar skeletal muscle composition.

**Methods:**

One hundred forty-one patients (78 females, mean age 57 ± 19 years) were prospectively enrolled and underwent lumbar spine MRI, EIM with Skulpt®, and clinical evaluation including the questionnaire SARC-F. MRIs were reviewed to assess the Goutallier score of paravertebral muscles at L3 level and to calculate the cross sectional area (CSA) of both psoas, quadratus lumborum, erector spinae, and multifidus muscles on a single axial slice at L3 level, in order to calculate the skeletal muscle index (SMI=CSA/height^2^). We tested the correlation between EIM-derived parameters [body fat percentage (BF%) and muscle quality] and body mass index (BMI), Goutallier score (1–4), SMI, and SARC-F scores (0–10) using the Pearson correlation coefficient. The strength of association was considered large (0.5 to 1.0), medium (0.3 to 0.5), small (0.1 to 0.3).

**Results:**

Pearson’s correlation coefficient showed small (0.26) but significant (*p* < 0.01) positive correlation between BF% obtained with EIM and Goutallier score. Small negative correlation (− 0.22, *p* < 0.01) was found between EIM muscle quality and Goutallier Score. Large negative correlation (− 0.56, *p* < 0.01) was found between SMI and Goutallier Score, while SMI showed small negative correlation with SARC-F (− 0.29, *p* < 0.01). Medium positive correlation was found between Goutallier Score and SARC-F (0.41, *p* < 0.01). BMI showed medium positive correlation with SMI (r = 0.369, *p* < 0.01) and small correlation with EIM muscle quality (r = − 0.291, *p* < 0.05) and BF% (r = 0.227, *p* < 0.05). We found a substantial increase of the strength of associations of BF% and muscle quality with Goutallier in the 18–40 years (r = 0.485 and r = − 0.401, respectively) and in the 41–70 years group (r = 0.448 and r = − 0.365, respectively).

**Conclusions:**

Muscle quality and BF% measured by EIM device showed only small strength of correlation with other quantitative parameters for assessing muscle mass and fat infiltration. Interesting results have been found in younger patients, but Skulpt Chisel™ should be applied cautiously to assess lumbar skeletal muscle composition. This point deserves further investigation and other studies are warranted.

**Trial registration:**

The registration number of this study is 107/INT/2019.

## Background

Sarcopenia is a condition defined by progressive loss of skeletal muscle mass and strength, which is associated to increased morbidity and mortality related to physical disability with possible risk of falls and fractures [1]. This condition, recently recognized as a muscle disease in the ICD-10-MC Diagnosis Code, have been widely investigated by several studies with increasing interest by the scientific community, being considered as an established independent unfavorable predicting factor for several disorders, including cancers, chronic diseases, trauma, infections [2–5]. From a clinical perspective, the SARC-F questionnaire is commonly used in clinical practice to diagnose sarcopenia. This questionnaire is a simple and well-established test recommended by the EWGSOP to identify patients with impaired physical function and sarcopenia, generally used to predict the risk of physical limitations and mortality [6–8]. Among the different imaging techniques that can be used to assess muscle status, dual-energy X-ray absorptiometry (DXA) is the one routinely used in clinical practice for its simplicity of use, despite computed tomography (CT) and magnetic resonance imaging (MRI) still are the most accurate techniques and are commonly used in research studies to estimate body composition using a single cross-sectional image [9]. In this setting, bioelectrical impedance analysis (BIA) is a non-invasive technique that uses an electrical current to measure conductance and resistance of body tissues for estimating body composition [10]. A recently introduced BIA device, namely Skulpt Chisel™, allows every subject to perform their own measurements of muscle quality and body fat percentage (BF%) on 24 different anatomical sites using Bluetooth™ technology to save and monitor all measurements with smartphones [11]. Some interesting results have been reported concerning the accuracy of this electrical impedance myography (EIM) device with comparative studies including DXA. However, DXA provides just a whole-body estimation of lean mass, while CT and MRI can measure muscle size and fat infiltration in specific districts [9]. Further, muscle and fat measurements from a single cross-sectional image allow to accurately estimate body composition, with a strong correlation between single-image and whole-body fat tissue and skeletal muscle distribution [12]. Moreover, CT and MRI are used for several diagnostic purposes, thereby being perfect to opportunistically evaluate sarcopenia without any additional examinations, with the latter imaging modality having the advantage of no radiation exposure. No previous studies have investigated the correlation of EIM measurements with MRI parameters and clinical characteristics. Thus, the aim of our study was to compare the degree of correlation between EIM parameter of body composition by Skulpt Chisel™ and MRI, in the specific assessment of lumbar skeletal muscle composition.

## Methods

This is a prospective study approved by our Institutional review board (registration number 107/int/2019 dated 20th June 2019, approved by Comitato Etico Ospedale San Raffaele). All patients who were enrolled provided written informed consent for collection of data to be used for scientific purposes. After matching imaging and clinical data, our database was anonymized to remove any connections between data and patients’ identity according to the General Data Protection Regulation for Research Hospitals.

### Patients enrollment

In this monocentric cross-sectional study, we evaluated a consecutive series of patients subjected to lumbar MRI from September 2019 to August 2022 at the IRCCS Istituto Ortopedico Galeazzi (Milan, Italy), a tertiary referral Orthopedic Centre, for diagnostic reasons independent of the study. All patients routinely subjected to lumbar MRI for assessing degenerative spine conditions were invited to participate to this study to compare lumbar skeletal muscle mass composition obtained with MRI to that assessed through EIM using the Skulpt Chisel™ device. After MRI and written informed consent to participate to this study, clinical evaluation was performed by a radiologist immediately after MRI, including data collection (age, gender, height, weight, and body mass index [BMI]), the SARC-F questionnaire for quickly screen for sarcopenia (Table 1) and EIM to measure BF% and muscle quality. The SARC-F questionnaire evaluates five features: strength, assistance in walking, rise from a chair, climb stairs, and falls. SARC-F scores range from 0 to 10 (0–2 points for each feature with 0 representing the best and 10 the worst) with a total score of ≥4 indicating sarcopenia [9]. We included patients with age over 17 who were able to understand and to provide written informed consent to undergo lumbar MRI and to participate to the study. The following exclusion criteria were applied: (i) clinical or psychiatric disorders that could hinder MRI or EIM performance; (ii) contraindications to MRI (such as claustrophobia, pacemakers or neurostimulators); (iii) history of cancer; and (iv) pregnancy. Setting α = 0.05, this yielded a sample size of 141 patients. Sample size was calculated using G*power software (v. 3.1.9.2, Dusseldorf University, Germany) [13].

**Table 1:** SARC-F questionnaire

Feature	Question	Answer
*Strength*	How much difficulty do you have in lifting and carrying 10 lb.?	None = 0 Some = 1 A lot or unable = 2
*Assistance in walking*	How much difficulty do you have walking across a room?	None = 0 Some = 1 A lot, use aids, or unable = 2
*Rise from a chair*	How much difficulty do you have transferring from a chair or bed?	None = 0 Some = 1 A lot, or unable without help = 2
*Climb stairs*	How much difficulty do you have climbing a flight of 10 stairs?	None = 0 Some = 1 A lot, or unable = 2
*Falls*	How many times have you fallen in the past year?	None = 0 1–3 falls = 1 ≥4 falls = 2

### Lumbar MRI protocol and images analysis

**Fig. 1:**
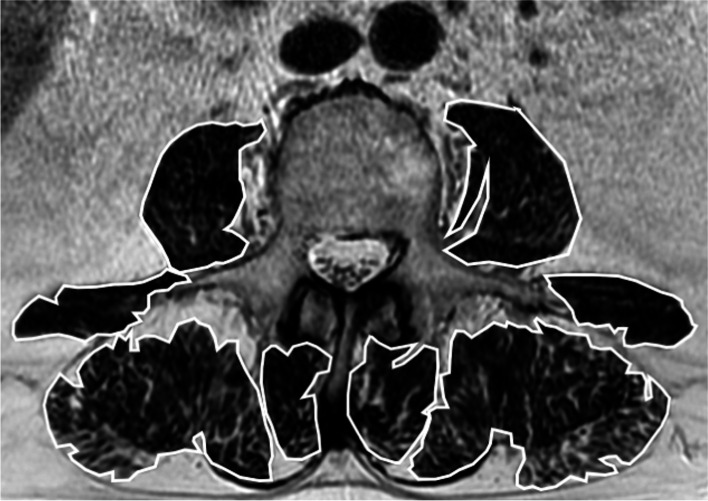
CSA and SMI. An example of manual segmentation from our study population with CSA values used to calculate SMI (CSA/height^2^)

**Fig. 2:**
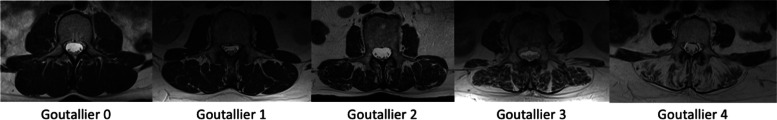
Goutallier classification system. Some cases with different Goutallier scores taken from our series. Goutallier score was 0 in 32 patients, 1 in 42, 2 in 54, 3 in 9, and 4 in 4

All lumbar spine MRI examinations were performed in two 1.5 T units (Avanto and Espree, Siemens, Erlangen, Germany, EU). MRI protocol included axial T2-weighted, sagittal T1-weighted, T2-weighted, and STIR images. A radiologist with 10 years of experience in musculoskeletal imaging and 5 years of experience in skeletal muscles segmentation, manually drew regions of interest to assess the cross sectional area (CSA) in mm^2^ of both psoas, quadratus lumborum, erector spinae, and multifidus muscles on a single slice obtained at the level of L3, where the transverse processes are well depicted [6]. CSA was used to calculate the skeletal muscle index (SMI) as follows: CSA/height^2^. Figure 1 shows an example of manual segmentation from our study population. The same radiologist reviewed axial T2-weighted images to perform a semiquantitative evaluation of fat infiltration in posterior paravertebral muscles using the Goutallier classification system [14]. The score was calculated using images from the midportion of the L4/L5 and L5/S1 intervertebral discs, given that there is the highest paravertebral muscles volume and relative percent fat at these levels [15]. This analysis was done in a different setting and 2 weeks later than manual segmentation. The Goutallier score was interpreted as follows: grade 0, normal muscle; grade 1, fatty streaks within the muscle; grade 2, fat less than muscle; grade 3, fat and muscle equal; and grade 4, fat greater than muscle. Figure 2 shows some cases with different Goutallier scores taken from our series.

### EIM

**Fig. 3:**
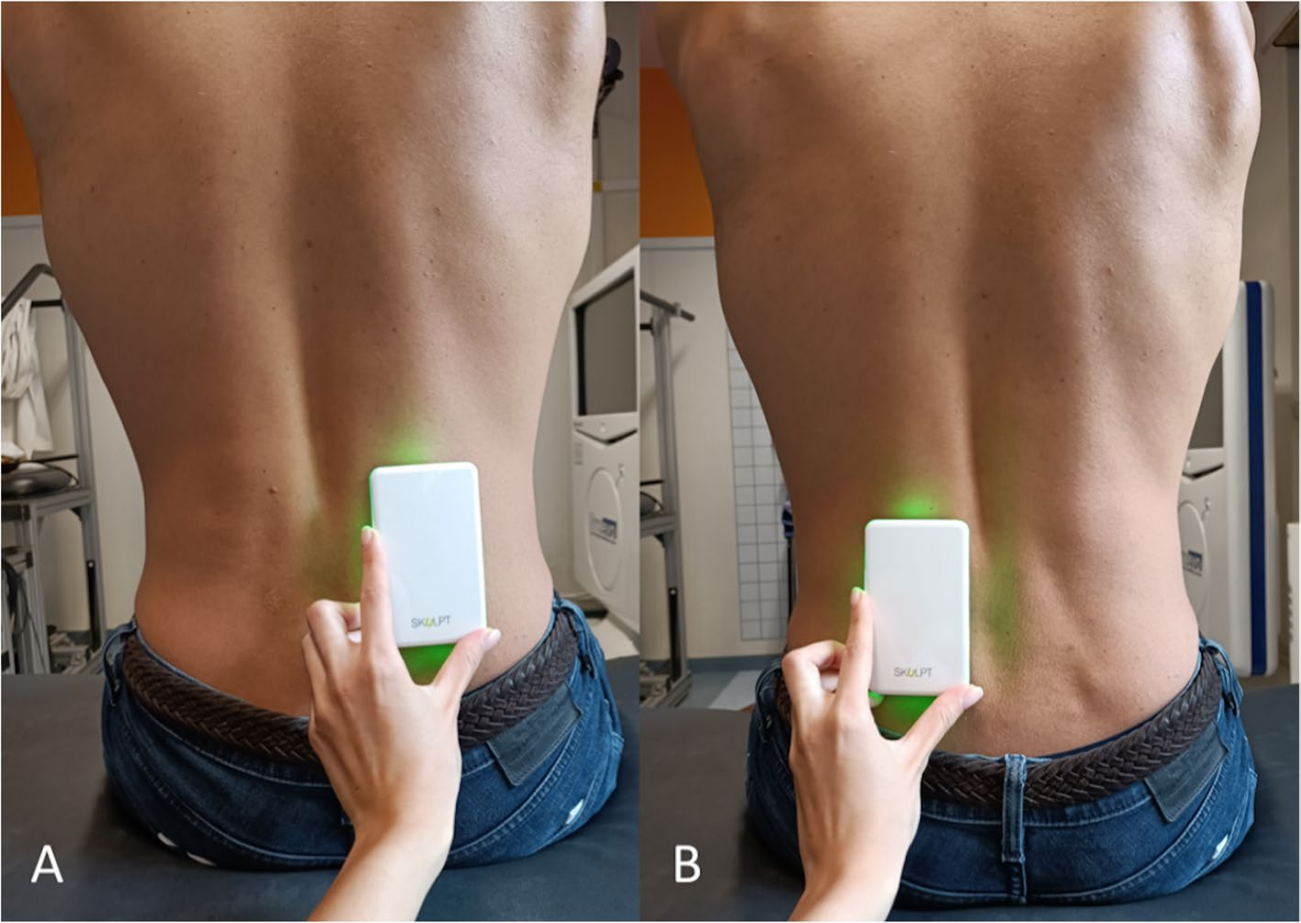
Skulpt Chisel™. An example of the correct placement of Skulpt EIM device in the right (**A**) and left (**B**) paravertebral muscles at L3-L4 level

BIA provides an estimation of skeletal muscle mass based on the application of an electric current across the human tissues and the measurement of current conduction [16]. This is possible due to the fact that muscles are the human tissues with the highest percentage of water. The Skulpt Chisel™ (Skulpt, Inc., Boston, Massachusetts, USA) was used to measure BIA. Skulpt Chisel™ is a portable device to perform EIM that, as BIA, is based on the evaluation of current conduction in human tissues to estimate body composition. The software allows to choose the muscle of interest and provides basic instructions for the use of the device. Before performing the lumbar measurements, a calibration was needed by applying the device to other muscles. The patient was examined while seated and normal saline was applied to the lumbar skin over the L3-L4 paraspinal regions using a wipe (Fig. 3). The electrode array was then applied to the skin on one side and three subsequent measurements were taken. Then, the same was done on the contralateral side. Data from triplicate measurements were averaged as a single entry in our analysis [17]. Final data obtained for each patient included BF% (a measure of body fat percentage) and muscle quality, with the latter ranging from 0 (worst) to 100 (best).

### Statistical analysis

Categorical variables were reported as absolute values and percentages, and continuous variables were reported as means ± standard deviations or as medians and interquartile ranges according to their distribution. Normality of data was evaluated using the Shapiro-Wilk test. The Spearman correlation coefficient was used to analyze the correlation between muscle quality and BF% obtained from EIM with BMI, Goutallier score (ranging from 1 to 4), SMI, and SARC-F scores (ranging from 0 to 10) using the Pearson correlation coefficient. We also tested the correlation of MRI data with SARC-F scores. Then, we repeated the correlation analysis after having divided all patients in three different groups according to their age (18–40 years, 41–70 years, over 70 years). Strength of Association was considered as follows: large (0.5 to 1.0), medium (0.3 to 0.5), or small (0.1 to 0.3). Statistical analysis was performed using SPSS® software v.24 (SPSS Inc., Chicago, IL, USA). A *p* value < 0.05 was considered as statistically significant.

## Results

One-hundred-forty-one patients were prospectively enrolled and underwent lumbar spine MRI, EIM with Skulpt®, and clinical evaluation including the questionnaire SARC-F. Of them, *n* = 78 (55%) were females and *n* = 63 (45%) were males. Goutallier score was 0 in *n* = 32 (23%) patients, Goutallier 1 in *n* = 42 (30%), Goutallier 2 in *n* = 54 (38%), Goutallier 3 in *n* = 9 (6%), and Goutallier 4 in n = 4 (3%). Ninety-seven (69%) patients (48 females, 49 males) presented SARC-F scores < 4, while the remaining 44 (31%) patients (30 females, 14 males) had SARC-F scores ≥4 consistent with sarcopenic status. Full demographic, MRI, and EIM data of our patients are resumed in Table 2.

**Table 2:** Demographic, MRI, and EIM data of 141 patients included in our study population

Age	57 ± 19 years (range 19–86)
Weight	74 ± 14 kg (range 50–98)
Height	170 ± 9 cm (range 153–192)
BMI	26 ± 3.9 (range 18–36)
Goutallier	1 (IQR:1–2, range 0–4)
SMI	2347 ± 5552 (range 1304–3949)
Muscle quality	47 ± 22 (range 10–99.3)
BF%	23 ± 6% (range 8.5–45.9)
SARC-F	2 (IQR = 0–4, range 0–8)

*BMI* Body mass index, *SMI* Skeletal muscle index, *BF%* Body fat percentage, *IQR* Interquartile range (1–3)

Pearson’s correlation coefficient showed small (r = 0.26) but significant (*p* < 0.01) positive correlation between BF% obtained from EIM and Goutallier score. Small negative correlation (r = − 0.22, *p* < 0.01) was found between EIM muscle quality and Goutallier Score. Large negative correlation (r = − 0.56, *p* < 0.01) was found between SMI and Goutallier Score, while SMI showed small negative correlation with SARC-F (r = − 0.29, *p* < 0.01). Medium positive correlation was found between Goutallier Score and SARC-F (r = 0.41, *p* < 0.01). BMI showed medium positive correlation with SMI (r = 0.369, *p* < 0.01) and small correlation with EIM muscle quality (r = − 0.291, *p* < 0.05) and BF% (r = 0.227, *p* < 0.05). All correlations of BF% and muscle quality obtained from EIM with Goutallier score, SMI, SARC-F scores, and BMI using the Pearson correlation coefficient are reported in Table 3.

**Table 3:** Correlations of BF% and muscle quality obtained from EIM with Goutallier score, SMI, SARC-F scores, and BMI using the Pearson correlation coefficient

		*BF%*	*SMI*	*Goutallier*	*SARC_F*	*M. quality*	*BMI*
*BF%*	r	1	− 0.116	0.257**	−0.014	− 0.788**	0.227
	p		0.236	0.002	0.865	0	0.019
*SMI*	r	−0.116	1	−0.565**	−0.289*	0.082	0.369**
	p	0.236		0	0.003	0.403	0
*Goutallier*	r	0.257**	−0.565**	1	0.409**	−0.224**	0.154
	p	0.002	0		0	0.008	0.114
*SARC-F*	r	0.014	−0.289*	0.409**	1	0.05	0.182
	p	0.865	0.003	0		0.556	0.061
*M. quality*	r	−0.788**	0.082	− 0.224**	0.05	1	−0.284*
	p	0	0.403	0.008	0.556		0.003
*BMI*	r	0.227*	0.369**	0.154	0.182	−0.284*	1
	p	0.019	0	0.114	0.061	0.003	

*M. quality* Muscle quality obtained from EIM, *BF%* Body fat percentage obtained from EIM, *SMI* Skeletal muscle index, *r* Pearson correlation coefficient, *p* significance; * = significant values with *p* < 0.05; ** = significant values with *p* < 0.01

Concerning the analysis of the different groups of patients according to their age, we found a substantial increase of the strength of associations of both BF% and EIM muscle quality with Goutallier score in the 18–40 years (r = 0.485 and r = − 0.401, respectively) and in the 41–70 years group (r = 0.448 and r = − 0.365, respectively). We also observed an interesting improvement of the association with the SARC-F score in the 18–40 years group (BF% r = − 0.422; muscle quality r = 0.317), with BMI in the 41–70 years group (BF% r = 0.618; muscle quality r = − 0.704), and with SMI in over 70 years patients (BF% r = − 0.446). All correlations of tested parameters in the three different groups of patients are reported in Table 4.

**Table 4:** Correlations of BF% and muscle quality obtained from EIM with Goutallier score, SMI, SARC-F scores, and BMI in three different groups of patients according to their age (18–40 years, 41–70 years, over 70 years) using the Pearson correlation coefficient

		*BF%*	*Goutallier*	*SARC-F*	*M. quality*	*SMI*	*BMI*
**18–40 yo**							
*BF%*	r	1	.485**	-.422**	-.785**	0.092	−0.006
	p		0.002	0.007	0	0.599	0.972
*Goutallier*	r	.485**	1	−0.086	-.401*	− 0.271	0.257
	p	0.002		0.603	0.011	0.116	0.136
*SARC-F*	r	-.422**	−0.086	1	.317*	−0.067	0.122
	p	0.007	0.603		0.049	0.701	0.485
*M. quality*	r	-.785**	-.401*	.317*	1	0.042	0.137
	p	0	0.011	0.049		0.812	0.434
*SMI*	r	0.092	−0.271	−0.067	0.042	1	.607**
	p	0.599	0.116	0.701	0.812		0
*BMI*	r	−0.006	0.257	0.122	0.137	.607**	1
	p	0.972	0.136	0.485	0.434	0	
**41–70 yo**							
*BF%*	r	1	.448**	0.16	-.771**	−0.014	.618**
	p		0	0.235	0	0.937	0
*Goutallier*	r	.448**	1	.412**	-.365**	-.371*	0.063
	p	0		0.001	0.005	0.026	0.717
*SARC-F*	r	0.16	.412**	1	0.036	-.429**	0.034
	p	0.235	0.001		0.791	0.009	0.842
*M. quality*	r	-.771**	-.365**	0.036	1	−0.249	-.704**
	p	0	0.005	0.791		0.142	0
*SMI*	r	−0.014	-.371*	-.429**	−0.249	1	.510**
	p	0.937	0.026	0.009	0.142		0.001
*BMI*	r	.618**	0.063	0.034	-.704**	.510**	1
	p	0	0.717	0.842	0	0.001	
**Over 70 yo**							
*BF%*	r	1	−0.118	−0.118	-.816**	-.446**	0.006
	p		0.439	0.439	0	0.006	0.971
*Goutallier*	r	−0.118	1	1000**	0.221	-.643**	−0.114
	p	0.439		0	0.144	0	0.507
*SARC-F*	r	−0.118	1000**	1	0.221	-.643**	−0.114
	p	0.439	0		0.144	0	0.507
*M. quality*	r	-.816**	0.221	0.221	1	0.236	−0.204
	p	0	0.144	0.144		0.166	0.232
*SMI*	r	-.446**	-.643**	-.643**	0.236	1	.414*
	p	0.006	0	0	0.166		0.012
*BMI*	r	0.006	−0.114	−0.114	−0.204	.414*	1
	p	0.971	0.507	0.507	0.232	0.012	

*M. quality* Muscle quality obtained from EIM, *BF%* Body fat percentage obtained from EIM, *SMI* Skeletal muscle index, *yo* years old, *r* Pearson correlation coefficient, *p* significance; * = significant values with *p* < 0.05; ** = significant values with *p* < 0.01

## Discussion

Our main finding is the small correlation we found between muscle parameters obtained by Skulpt Chisel™ and demographic characteristics, SARC-F scores, as well as with MRI parameters of lumbar spine muscles composition.

To the best of our knowledge, this is the first study to examine the correlation of lumbar BF% and muscle quality obtained by EIM with MRI quantitative and semiquantitative parameters of sarcopenia and myosteatosis. Previous papers have shown interesting results when comparing EIM and DXA data. Mclester et al. reported no significant differences (*p* > 0.05) between BF% obtained by EIM and DXA, as well as by BIA and skinfold measures [18]. The authors underlined that Skulpt Chisel™ might enable to correctly estimate BF% similar to DXA but through a quick and non-invasive way using a handheld EMI device, performing better than the widely used field method of skinfolds with smaller standard error of the estimates, total errors, and narrow limits of agreement. Of note, their subjects were young healthy volunteers that were supposed to have healthy BF% values. Conversely, we enrolled patients performing MRI for assessing spine disorders and variable ages, including older sarcopenic subjects. Further, it should be taken into account that EIM evaluation can be strongly affected by the hydration status of lean mass, given that hyperhydration may lead to underestimation of BF% and vice versa, with conditions of altered hydration being more frequent in elderly [19]. These findings can partly justify our small correlation between Skulpt Chisel™ and other quantitative parameters for muscle composition. Notably, Czeck et al. reported significantly lower regional BF% measured by Skulpt Chisel™ than DXA, particularly for upper left arm, upper right arm, upper right leg, and trunk (*p* < 0.003), although no significant differences were observed in total BF% (*p* = 0.434) [20]. Their results may partly explain why Skulpt Chisel™ data poorly correlate with regional lumbar muscle status of our subjects identified by MRI, since at a first glance this EIM device appears to be a reliable tool to assess global BF%, as previously reported by other authors too [18, 21]. Some limits of Skulpt Chisel™ device have been highlighted also by Wells et al. that reported significant difference in BF% when comparing EIM values with seven-site skinfold and hydrostatic weighing BF% estimates (*p* < 0.05) [11]. In our study, we used two imaging parameters to investigate the correlation of EIM device with MRI parameters, specifically the SMI and Goutallier scores. MRI accuracy in evaluating CSA on cross-sectional images has been shown to be very high, with almost perfect correlation with CT (up to r = 0.99) [12, 22]. CSA is used to obtain the SMI with cut-off values standardized by meta-analysis studies to differentiate sarcopenic men and women [23]. Indeed, while DXA provides an estimation of total body lean mass, CT and MRI axial images can be used to obtain measurements from specific muscles that have shown to be highly accurate in body composition estimation [9, 12, 24]. Concerning the Goutallier score, it is a semiquantitative classification CT system adapted to MRI to assess fat infiltration in muscle bellies. We acknowledge that the best would be to evaluate fat infiltration on axial T1-weighted images, but we preferred to use axial T2-weighted that are routinely included in spine MRI protocols, after checking sagittal STIR images to exclude any patient with edema in the paravertebral muscles. Notably, the subgroup analysis according to patients’ age highlighted that the association of EIM-derived parameters and Goutallier score might be probably higher than that showed by the analysis on our whole series. Indeed, medium correlations of BF% and muscle quality were found in the 18–40 years and 41–70 years groups of patients. As a matter of fact, the dramatic decrease of association in the over 70 group was related to the fact that almost all over 70 years patients had the same Goutallier score (42/45 patients with Goutallier 3 and 2/45 patients with Goutallier 2), thereby affecting the results of our statistical analysis. This might partly justify the small correlations found with Goutallier in the whole group of patients. Of note, a limitation of our study is that manual segmentation of lumbar muscles and Goutallier scores were provided by a single experienced reviewer, but it has been proven that these measurements are strongly reliable and reproducible, being widely used for research purposes [9, 14, 24].

Regarding clinical characteristics of our patients, we found small to medium correlations of SARC-F scores with SMI and Goutallier score, respectively, while no significant correlations were found with EIM parameters. On the other hand, both EIM-derived muscle quality and BF% showed medium correlations with SARC-F scores in the younger patients of our series. Nevertheless, it should be noted that SARC-F questionnaire is a helpful tool to screen for low muscle strength and physical performance, but it has well-known limitations, especially screening ability for identifying patients with sarcopenia [1, 25, 26].

## Conclusions

In conclusion, muscle quality score and BF% measured by EIM device showed only small degrees of correlation with other quantitative parameters for measuring muscle mass and fat infiltration. Interesting results have been found in younger patients, although our data drive us to state that Skulpt Chisel™ should be applied cautiously to assess lumbar skeletal muscle composition. This point deserves further investigation and other studies are warranted to test the correlation between EIM values and imaging parameters in other anatomic districts.

## Data Availability

All data are fully available upon reasonable request. The corresponding author should be contacted if someone wants to request the data.

## Abbreviations

DXA: Dual-energy X-ray absorptiometry
CT: Computed tomography
MRI: Magnetic resonance imaging
BIA: Bioelectrical impedance analysis
BF%: Body fat percentage
EIM: Electrical impedance myography
BMI: Body mass index
CSA: Cross sectional area
SMI: Skeletal muscle index
